# Commentary: Non-pathogenic *Neisseria* species of the oropharynx as a reservoir of antimicrobial resistance: a cross-sectional study

**DOI:** 10.3389/fcimb.2023.1343608

**Published:** 2024-01-09

**Authors:** Chris Kenyon

**Affiliations:** ^1^ Department of Clinical Sciences, Institute of Tropical Medicine Antwerp, Antwerp, Belgium; ^2^ Department of Medicine, University of Cape Town, Cape Town, South Africa

**Keywords:** commensal *Neisseria*, AMR (antimicrobial resistance), antimicrobial consumption monitoring, *Neisseria gonorhoeae*, Italy

## Introduction

In this issue, Gaspari et al. report results of the first survey of antimicrobial resistance (AMR) in commensal *Neisseria* spp. in Italy (Gaspari et al., 2023). They found a high prevalence of ceftriaxone resistance in oral commensal *Neisseria* spp. (11.7%: breakpoint >0.125mg/L), which did not differ between the general population and men who have sex with men (MSM). This prevalence is very similar to that found in a similar study design in Belgium by Laumen et al. (2022), from 2022, where the prevalence of resistance was 6.9% (same breakpoint), and again did not differ between the MSM and the general population. To the best of our knowledge only one other country (Vietnam) has performed an analogous survey. This was a study in 2029 by Dong et al., who found that the prevalence of ceftriaxone resistance (same breakpoint) in commensal Neisseria was considerably higher (28%) than that found in Belgium or Italy (Dong et al., 2019).

As Gaspari et al. note, horizontal gene transfer of the *penA* and other genes from commensal *Neisseria* spp. to *N. gonorrhoeae* has been an important determinant of gonococcal reduced susceptibility to cephalosporins and other antimicrobials (Manoharan-Basil et al., 2021; Goytia and Wadsworth, 2022). This has led to the proposal that surveillance of AMR in commensal *Neisseria* could be used as an early warning system of excessive antimicrobial consumption in a population that puts them at risk for AMR (Kenyon et al., 2021; Goytia and Wadsworth, 2022). This proposal depends on the existence of a positive association between antimicrobial consumption and AMR in commensal *Neisseria* spp. As yet, no study has assessed if such an association exists.

## Association between cephalosporin consumption and resistance

The data from these three studies in Belgium, Italy and Vietnam offer us the opportunity to test this hypothesis. To do this we used Spearman’s correlation to test the association between the prevalence of ceftriaxone resistance in commensal *Neisseria* spp. and the consumption of cephalosporins in the population. The data for the cephalosporin consumption was taken from IQVIA (IQVIA, Danbury, CT). IQVIA uses national sample surveys that are performed by members of pharmaceutical sales distribution channels to estimate antimicrobial consumption from the volume of antibiotics sold in retail and hospital pharmacies (Klein et al., 2018). Antimicrobial consumption estimates are reported as the number of standard doses per 1000 population per year (DDD). We used data from 2015 which is the most recent year with data available (Klein et al., 2018).

**Figure 1:**
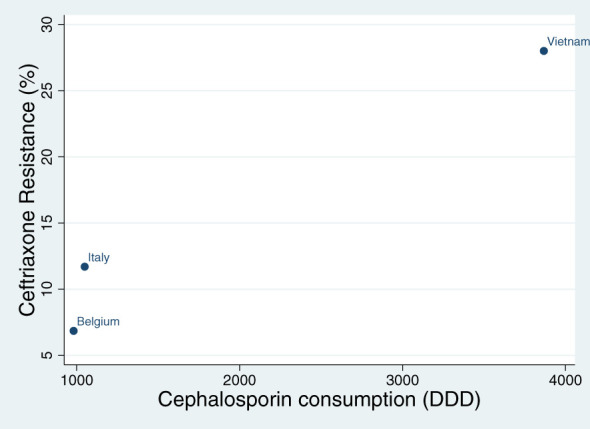
Spearman’s correlation of the association between cephalosporin consumption (defined daily doses per 1000 persons – DDD) and the prevalence of ceftriaxone resistance in commensal *Neisseria* species in Belgium, Italy and Vietnam (Rho 1; P<0.001).

The consumption of cephalosporins varied between 981 DDD in Belgium, 1049 DDD in Italy and 3867 DDD in Vietnam. Spearman’s correlation revealed a positive correlation between cephalosporin consumption and the prevalence of ceftriaxone resistance in each country (Rho 1; P<0.001; **Figure 1**).

## Discussion

An important limitation of this analysis is that there were small differences between the studies in terms of setting, selection of participants, sample collection and culturing of commensal *Neisseria*. All three studies did however obtain oropharyngeal samples with a swab, identified the isolates via oxidase testing and MALDI-TOFF and used Etests to determine the MIC. There was a slightly higher proportion of isolates that were *N. perflava* in Vietnam than the other two countries. Ceftriaxone susceptibility was not, however, found to vary between the commensal *Neisseria* species in any of the studies (Gaspari et al., 2023; Dong et al., 2019; Laumen et al., 2022). In addition, there may have been a difference in the prevalence of *Neisseria* spp. carriage between the three studies. Furthermore, the use of Spearman’s correlation is a crude measure of cross-sectional association that does not take into account variations in antibiotic consumption and resistance over time.

These limitations notwithstanding, this positive association provides evidence that the prevalence of resistance in commensal *Neisseria* spp. may be a useful early warning system of excessive antimicrobial consumption in a population. It would be very useful for future studies to expand these results to include populations with lower antimicrobial consumption, a broader range of antimicrobials and compare commensal *Neisseria* with other genera such as oral streptococci as optimal sentinel genus. Finally, it will be important to establish if reducing antimicrobial consumption is followed by a reduced prevalence of AMR.

## Author contributions

CK: Writing – original draft, Writing – review & editing.
